# Angiosarcoma: A Rare Malignancy Linked to Chemical Exposures

**DOI:** 10.7759/cureus.25289

**Published:** 2022-05-24

**Authors:** Sophia T Tessema, Abdullahi E Mahgoub, Rasha Nakhleh

**Affiliations:** 1 Neurology, Wayne State University, Detroit, USA; 2 Internal Medicine, Michigan State University / Hurley Medical Center, Flint, USA; 3 Internal Medicine & Geriatrics, Oregon Health & Science University, Portland, USA

**Keywords:** geriatric medicine, medical dermatology, older adult, epithelioid angiosarcoma, rare cancers

## Abstract

Angiosarcoma is an exceptionally rare malignancy that accounts for less than 1% of all sarcomas. This case describes a 90-year-old male veteran who presented with a hematoma from a traumatic head injury. This then progressed to an ulcerated bleeding lesion that measured 2.5 cm in diameter with pearly borders and granulation tissue. CT scan of the head and skin biopsy were consistent with the diagnosis of cutaneous angiosarcoma. The patient may have unique exposures from the military training site Camp Lejeune including tetrachloroethylene (PCE), trichloroethylene (TCE), trans-1,2-dichloroethylene, and vinyl chloride predisposing to angiosarcoma. The patient underwent palliative radiation without obvious complications. This case presents an opportunity for further evaluation and understanding of the effects of these exposures and the implications for the health of veterans and aging populations. Patient outcomes may be improved with earlier diagnosis and aggressive treatment.

## Introduction

Angiosarcoma is an exceedingly rare, aggressive malignancy accounting for less than 1% of all sarcomas [1,2]. Most of the data pertaining to angiosarcomas is derived from samples of less than 100 patients in cohort studies or case reports [1]. Studies have suggested less than 20 cases of angiosarcoma of the skull were reported worldwide by 2013 [3]. The prognosis is poor; some studies point to a 12% five-year mortality rate [4]. The majority of sarcomas are in deep tissues; however, angiosarcomas have the propensity to appear in the skin and superficial soft tissue, with the scalp comprising 50% of all angiosarcomas [1]. Nonetheless, angiosarcoma of the scalp contributes to less than 0.1% of all head and neck malignancies [1]. The population at greatest risk of this cancer are elderly Caucasian adults, with a male to female ratio of 3:1 [5]. Patients present with an enlarging bruise, a discolored nodule, or persistent ulceration. In the early stages, these lesions can be misdiagnosed as benign entities caused by cellulitis, infection, herpes zoster, or skin injuries [1,6]. Risk factors for the development of angiosarcoma include radiation exposure, familial syndromes, and, more recently, chemical exposures [1,4,7]. Our case presents a patient with unique exposures due to training at the Base Camp Lejeune in North Carolina, USA.

Veterans face unique challenges as a result of their training and service, including mental health disorders, substance use disorders, debilitating injuries, and cancer. Approximately 40,000 new cancer cases in veterans are reported annually [8]. The incidence of cancer is likely the result of radiation and growing evidence linking cancer to herbicides and chemical weapons, particularly chemical exposure at Camp Lejeune [1,2,4,5].

Those who trained at the Marine Corps Base Camp Lejeune from 1957 through 1987 were exposed to over 3000 times the safe exposure limit of toxic chemicals through the contaminated water [7]. The chemicals were industrial solvents contaminating the water treatment systems supplying the camp. These included tetrachloroethylene (PCE), trichloroethylene (TCE), trans-1,2-dichloroethylene, and vinyl chloride, highly carcinogenic agents associated with adult leukemia, Parkinson’s disease, and bladder cancer, among other malignancies [7].

By presenting this case, we want to highlight the possible association between angiosarcoma of the skull and chemical exposure at Camp Lejeune.

## Case presentation

The patient is a 90-year-old Caucasian male with a past medical history of Alzheimer’s dementia, stage one squamous cell carcinoma of the lung treated with resection, chronic obstructive pulmonary disease, squamous cell carcinoma of the scalp, and a former smoker. Our patient is a veteran who served in the marine corps and trained for two years at Camp Lejeune.

**Figure 1:**
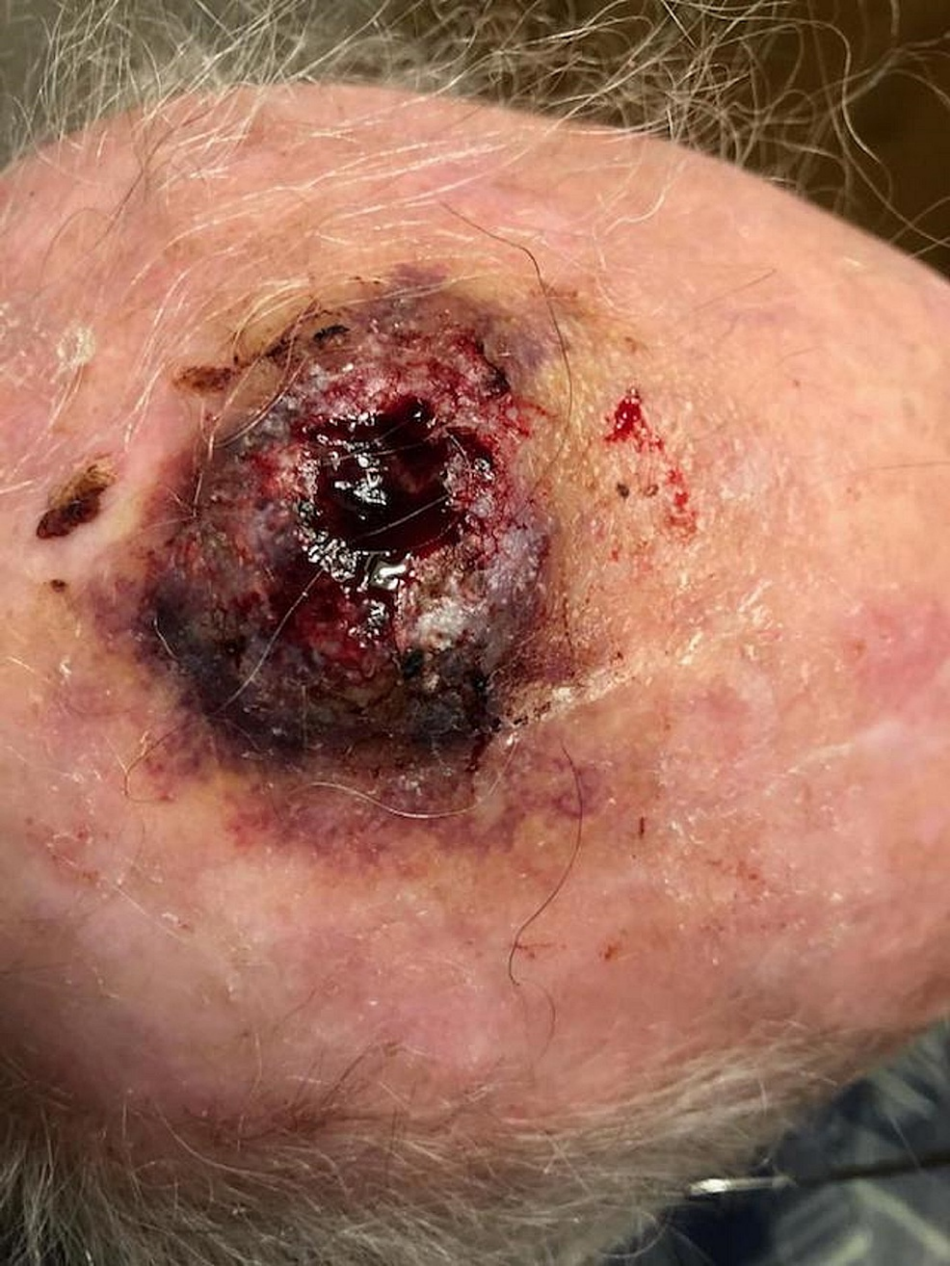
Non-healing ulcer with persistent bleeding

Our patient presented to the geriatric clinic after head trauma. He developed a bruise and a 3 cm hematoma. Approximately one month later, the patient was seen in urgent care because the site of scalp trauma began to bleed. He was prescribed cephalexin for suspected cellulitis without healing of the wound. One month later, he developed ulceration and bleeding, and a 2.5 cm diameter lesion with pearly borders and granulation tissue was noted. CT scan was performed and demonstrated soft tissue swelling in the superior posterior portion of the left frontal region with a 3.7 cm diameter and a maximum thickness of about 13 mm. The calvarium was intact, with no foreign bodies noted within the swelling. A skin biopsy was arranged due to the non-healing ulcer and persistent bleeding, raising suspicion for malignancy (Figure 1). The pathology report revealed angiosarcoma with a concurrent atypical squamoproliferative lesion concerning for invasive, well-differentiated squamous cell carcinoma extending to deep and peripheral margins.

Treatment

**Figure 2:**
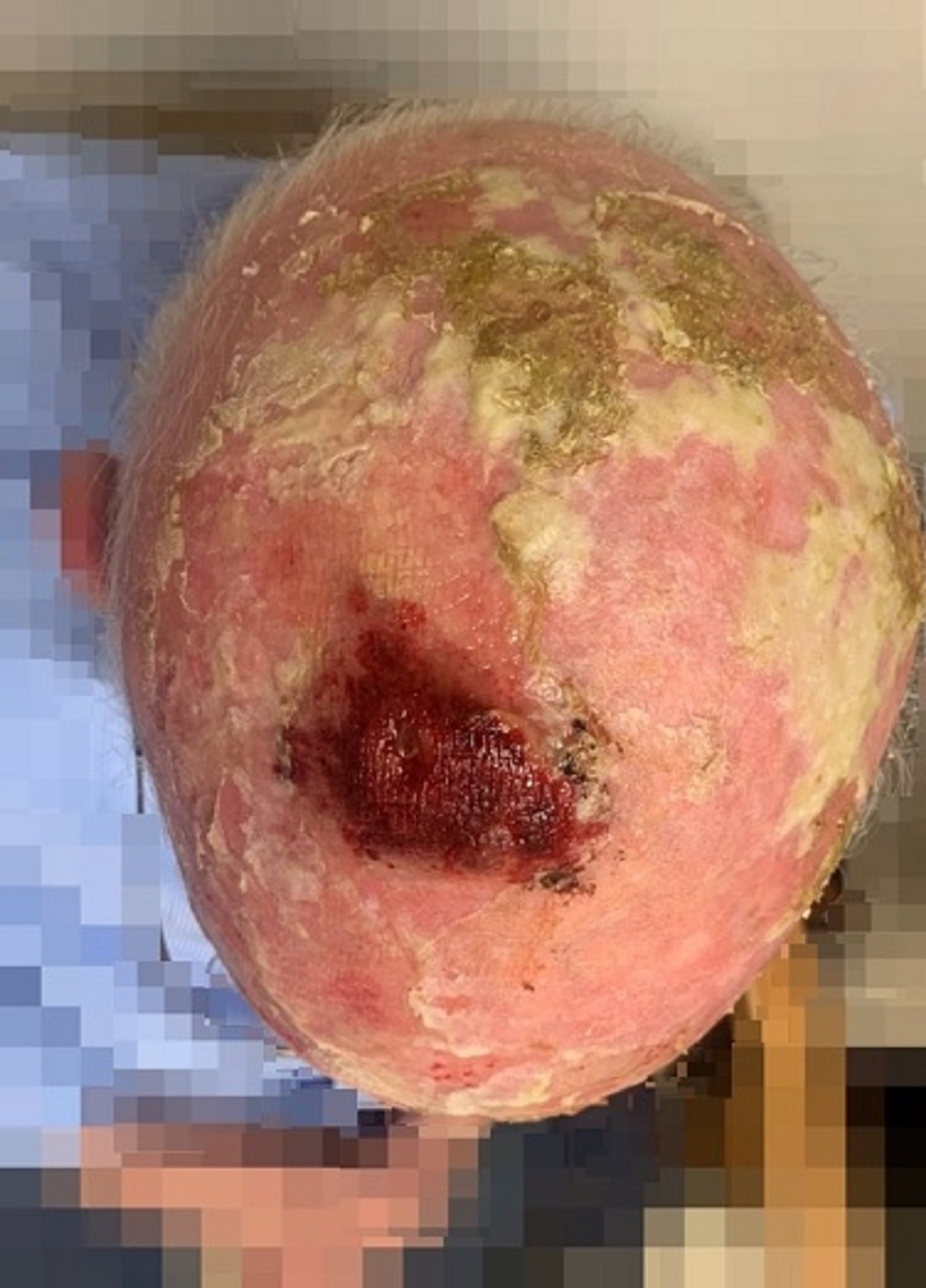
Ulcer during radiation therapy

**Figure 3:**
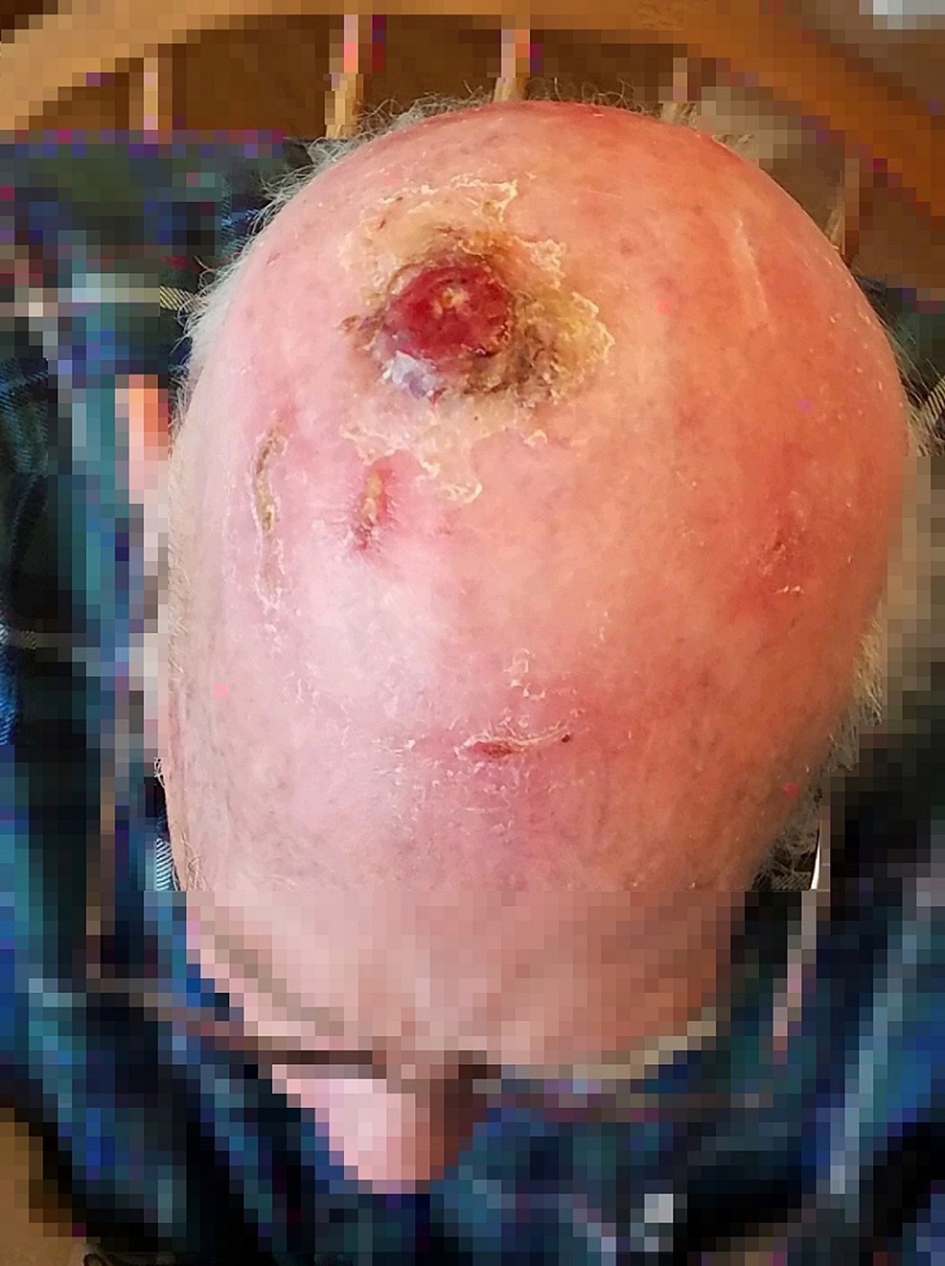
Ulcer post-radiation therapy

The patient underwent palliative radiation therapy for high-grade cutaneous angiosarcoma of the scalp without obvious complications (Figure 2, 3).

Outcome and follow-up

Six months after diagnosis with angiosarcoma, our patient passed away due to pneumonia complicated by pleural effusion and hemopneumothorax with ultimate hypoxic respiratory failure.

## Discussion

Angiosarcomas are rare and aggressive malignancies representing about 2% of all adult soft tissue sarcomas and comprise less than 1% of all head and neck malignancies [9, 10]. They are difficult to treat and have a poor prognosis [1].

Various risk factors may contribute to the development of angiosarcoma [1]. Trauma is not itself a risk factor, but it can serve as a catalyst to the diagnosis as injury to the tumor site can present with prolonged bleeding, as seen in our patient [11]. The patient and his wife were not able to recall repeated injury.

Smoking has not demonstrated an increased risk for angiosarcoma; however, his smoking history likely precipitated his squamous cell carcinoma of the lung [10,11]. The squamous cell carcinoma of the scalp is likely due to the nature of the patient's work on the horse farm from recurrent sunlight exposure, although the patient recalls always wearing a ball cap or cowboy hat outside. It is unclear if there is a relationship between squamous cell carcinoma and the development of angiosarcoma. 

Known risk factors for the development of angiosarcoma include hereditary syndromes, including neurofibromatosis type one, Klippel-Trenaunay syndrome, and Maffucci syndrome, among others [11]. Radiation is also a risk factor, however, the incidence of a radiation-associated sarcoma is estimated to be under 1% in exposed adult populations [1,2,5,7,11, 12]. Our patient did not have these hereditary diseases and did not receive radiation therapy for his squamous cell carcinoma. However, he did train at the Marine Base Camp Lejeune during the time period of the water contamination. 

According to the National Toxicology Program, the chemicals tetrachloroethylene (PCE), trichloroethylene (TCE), trans-1,2-dichloroethyelene, and vinyl chloride are known human carcinogens. These chemicals were found in toxic levels at the supply wells in the Base Camp Lejeune. A retrospective cohort study found an increased risk of mortality from cancers, including soft tissue cancers and other chronic diseases amongst marine and naval personnel exposed to these substances [7].

A study conducted by the Centers for Disease Control enrolled 247,479 participants from the Defense Manpower Data Center and the Agency for Toxic Substances and Disease Registry to compare Camp Lejeune to Camp Pendleton marines' mortality due to cancer and non-cancer cases [13]. The odds ratio for soft tissue cancer was 1.27, and for high residential exposures to TCE and PCE, the odds ratio was 1.33 [13]. These results support more cases of soft tissue cancer in the group exposed to the water contamination. In this study, there were two cases of soft tissue cancer at the Camp Lejeune site and none at the comparison site of Camp Pendleton. This study also found soft tissue cancer and TCE-related skin disease were reported only in men [13]. These findings support the epidemiologic evidence of a higher prevalence of soft tissue carcinoma among men as well as providing evidence of a likely link between soft tissue cancers, such as angiosarcoma, and contaminated water at Camp Lejeune. 

Currently, 15 health conditions are recognized for health benefits from the USA Department of Veterans Affairs (VA) for those who served at least 30 days of active duty at Camp Lejeune from August 1, 1953, through December 31, 1987 [14]. Further research on the relationship between water contamination at Camp Lejeune and the development of angiosarcoma may support the addition of angiosarcoma to the list of health conditions with covered benefits.

Additionally, further studies can elucidate the etiologies of angiosarcoma, which can aid in achieving better treatment of this condition, and ultimately improve the prognosis. Our case, along with the available literature, adds anecdotal information to the body of evidence of the health effects of these chemicals that should be evaluated in all veterans with such exposures.

## Conclusions

Angiosarcoma is a rare disease that can be difficult to diagnose based on initial presentation of bruise-like skin condition but should be considered in high-risk patients with this presentation. This case presents an opportunity for further evaluation and understanding of the effects of these exposures and the implications for the health of veterans and aging populations. Patient outcomes may be improved with earlier diagnosis and aggressive treatment. In addition, concomitant malignancies may delay reaching the final diagnosis due to overlapping symptoms. 

## Appendices

Patient's perspective

“The first bump or bruise was hardly noticeable. Two months later it became a concern when it didn’t heal and began to bleed continuously. A biopsy determined the cancer. Treatment of radiation was a piece of cake and made a significant improvement. At 90 years old I’m thankful to be alive and living the good life. Thank you all!”
